# The relationship between socioeconomic factors, social participation, behaviours, and healthy ageing: an analysis of longitudinal data

**DOI:** 10.3389/fpubh.2026.1932979

**Published:** 2026-09-30

**Authors:** Lujain Sahab, Tim Newton, Wael Sabbah

**Affiliations:** Faculty of Dentistry, Oral and Craniofacial Research, King’s College London, London, United Kingdom

**Keywords:** health and retirement study, healthy ageing, longitudinal study, older adults, social participation

## Abstract

**Objectives:**

To investigate the longitudinal associations between social participation, socioeconomic factors, health behaviours and Healthy Ageing (HA) among older Americans over a ten-year period.

**Methods:**

Retrospective secondary analysis using data from three waves of the Health and Retirement Study, corresponding with years (2006, 2010, 2016), a longitudinal survey of older American adults. The analysis included demographic factors (gender, age, race, marital status), and socioeconomic factors (education, total wealth and poverty-income ratio) in 2006, health behaviours (smoking, physical activity), and Body Mass Index in 2010, social participation in 2006 and HA (measured in 2010 and 2016). Healthy ageing was an aggregate variable including freedom from disability, freedom from cognitive impairment, and high physical functioning. Social participation was a dichotomous variable derived from work status, perceived neighbourhood safety, and engagement in volunteer work. Structural equation modelling (SEM) was employed to examine direct and indirect associations between these factors and HA at final follow-up.

**Results:**

A total of 9,748 participants with complete data in the three waves were included. The mean age at baseline was 68 years. Social participation in 2006 was significantly associated with HA in 2016 (estimate: 0.09, 95% CI: 0.08, 0.11). Physical activity showed a strong positive association with HA in 2016 (estimate: 0.31, 95% CI: 0.29, 0.33). Furthermore, HA in 2010 was also a strong exposure associated with HA in 2016 (estimate: 0.46; 95% CI: 0.45–0.47).

**Conclusion:**

Social participation was significantly associated with HA, alongside established health behaviours such as physical activity. These findings underscore the importance of social participation as a key factor associated with HA and support the integration of social participation into public health strategies for older adults.

## Introduction

Healthy ageing (HA), defined as the process of maintaining functional ability and wellbeing in older age, is a critical public health priority (1). As the global population ages, understanding the factors that contribute to HA becomes increasingly important (2). While biological and genetic factors play a role (3), an increasing number of studies emphasise the significant impact of social and economic factors on health in later life. An increasing number of studies emphasise the significant impact of social and economic factors on health in later life (4–6).

Contemporary systematic reviews and meta-analyses indicate that a wide range of sociodemographic and behavioural determinants, including educational attainment, income/wealth, social participation, and physical activity, are significantly associated with HA outcomes across diverse settings, highlighting the multifaceted nature of HA beyond biological influences (7, 8). In addition, evidence suggests that models of HA incorporating multidimensional frameworks better capture the complex interplay of physical, psychological, and social domains than unidimensional approaches (7). Embedding these findings within theoretical frameworks such as life-course and biopsychosocial models underscores the importance of social structures, economic conditions, and cumulative exposures across the lifespan in shaping health trajectories in later life, offering a comprehensive basis for research and policy aimed at promoting wellbeing in ageing populations (9).

Socioeconomic inequalities, manifested in education, income, and wealth, are particularly salient (10, 11). These inequalities can accumulate throughout life, producing substantial differences in access to resources, exposure to stressors, and opportunities for health-promoting behaviours, ultimately impacting HA trajectories (12, 13). Higher socioeconomic status is linked to better access to healthcare and preventive services, greater health literacy, and more resources that support health-promoting lifestyles, while lower socioeconomic status is associated with increased exposure to stressors and barriers to healthy behaviours, ultimately contributing to disparities in ageing outcomes. A systematic review of multidimensional HA measures found consistent positive associations between socioeconomic status indicators and healthier ageing across populations, underscoring the social gradient in ageing processes (14).

Health behaviours such as physical activity and smoking are among the determinants of HA, influencing biological pathways related to cardiovascular health, inflammation, metabolic regulation, and functional capacity. Patterns of these behaviours are socially patterned, with individuals of higher SES generally engaging in more health-promoting behaviours, such as regular physical activity and lower rates of smoking, compared with those of lower SES, which may partly mediate SES disparities in ageing outcomes (15, 16).

Aside from the role of socioeconomic factors, social participation, encompassing involvement in social activities, community organisations, and social networks, has been linked to multiple health benefits in older adults, involving better physical and mental health, reduced social isolation, and increased access to social support (17, 18). Social participation has been demonstrated to affect health in older adults, partly by encouraging healthier behavioural patterns. Social participation is associated with lower risks of physical inactivity, poor eating habits, and diminished intellectual activity (19). Highlighting the important role of social connections and community involvement in promoting health and wellbeing in ageing populations (17, 20).

Social participation among older adults has been identified as a crucial factor in extending the period of functional independence before death. Individuals who participate in any social groups are significantly less likely to experience accelerated or severe disability trajectories compared to those with minimal participation (21). Empirical evidence indicates that social participation is associated with better activities of daily living and self-rated health, partly operating through its influence on functional health trajectories (22, 23). Exploring these social pathways within a longitudinal framework can provide valuable insights into how social participation interacts with other determinants, such as socioeconomic status and health behaviours, to influence ageing trajectories.

Despite evidence linking socioeconomic status, social participation, and health behaviours to various dimensions of health in older adults, the longitudinal pathways through which these factors interact over time to influence HA remain less understood. To investigate these longitudinal relationships, this study used data from the Health and Retirement Study (HRS) collected in 2006, 2010, and 2016. These waves were selected based on data availability and consistency of measurement. The 2006 wave was chosen as the baseline because several variables required for this study were not available in earlier HRS waves. The 2010 wave was used to capture intermediate measures, including social participation and baseline healthy ageing, while the 2016 wave was selected as the final follow-up because the variables used to operationalise the HA outcome were not consistently collected in subsequent waves. This design ensured that all study variables were available and measured consistently across the study period, while maintaining the temporal ordering necessary to examine longitudinal pathways between socioeconomic factors, social participation, health behaviours, and healthy ageing.

Despite the rich conceptualisation of healthy ageing, existing research predominantly focuses on cross sectional associations or isolated health outcomes, with limited longitudinal investigations into the mediating pathways and interactions among social participation, socioeconomic factors, and health behaviours over extended periods. Few studies employ comprehensive modelling techniques, such as structural equation modelling, to disentangle direct and indirect effects across multiple domains. Consequently, there remains a significant gap in understanding how these factors interrelate dynamically over time to influence overall healthy ageing trajectories, particularly within diverse populations of older adults in the United States.

The study aimed to address these gaps in the literature by investigating the longitudinal associations between various factors (social participation, socioeconomic factors, health behaviours) and HA among older Americans over a ten-year period. By exploring these complex relationships, it contributes novel insights in understanding the social determinants of HA and support the development of targeted interventions to promote HA across all socioeconomic groups.

## Materials and methods

### Data source

The study extracted data from the Health and Retirement Study (HRS), a nationally representative longitudinal survey of American Adults aged 50 years and above. Data is collected biennially. Three waves were included in the current study: wave 8 (2006), wave 10 (2010), and wave 13 (2016). The Health and Retirement Study was approved by the Institutional Review Board. All participants provided informed consent prior to participation in the HRS. The present study involved secondary analysis of de-identified data no consent was needed.

### Study design and sample

The HRS follows a large cohort of adults across the United States, collecting data on health, demographic characteristics, and socioeconomic factors. In the present analysis, the final sample included 9,748 participants, drawn from three survey waves. Only individuals with complete data across all relevant domains over the ten-year follow-up period were included.

### Study variables

The analysis included a variety of exposure variables, including demographic factors, socioeconomic factors, and social participation. Demographic Factors included gender (male/female), age (continuous variable), and marital status classified into married, unmarried/divorced, widowed. Race sorted to two groups individuals being Black, non-Black.

Socioeconomic factors included education, total wealth, and income. Educational attainment was classified into four stages: less than high school, high school diploma, some college, and college degree or higher. Total wealth was expressed in nominal dollars and calculated as the sum of assets minus debts, as it was described in the HRS. For this analysis, wealth was categorised into quartiles (lowest to highest). The poverty-income ratio, indicated as “income” in the text, represented the ratio of annual household income to the US Census poverty thresholds. Similar to wealth, income was divided into quartiles.

Social participation was conceptualised as a multifaceted construct that encompassed three key factors. This composite measure was used based on previous literature (24), including the successful ageing framework proposed by Rowe and Kahn (25), has operationalised social participation as a multidimensional construct incorporating engagement in productive, social, and community activities rather than relying on a single indicator. The measure used in the present study was therefore based on an established approach and allowed different forms of societal engagement to be captured within a single indicator of social participation. These factors included individuals’ work status, perceived neighbourhood safety, and involvement in volunteer work. Individuals were asked if they were doing any work for pay (0 = no, 1 = yes), if they perceived their neighbourhood safety as fair/poor (0) or excellent/very good/good (1), and if they had spent any time in the past 12 months doing volunteer work for religious, educational, health-related, or other charitable organisations (yes/no). Finally, the variable was dichotomised (yes/no); individuals were considered to participate in their society if all previous conditions were met.

Behavioural factors considered in this study comprised of physical activity and smoking. Physical activity was measured according to both the frequency and intensity of various activities the measure included a combination of three variables found in the study (vigorous, moderate, and mild physical activity). The intensity of the activity was determined by the type performed. Vigorous physical activity included activities such as running or jogging, swimming, cycling, aerobics or gym workout, tennis, or digging with a spade or shovel.

Moderate activity categorised as gardening, cleaning the car, walking at a moderate pace, dancing, floor or stretching exercises. While mild activity included vacuuming, laundry, and home repairs. Frequency of said activities were also documented as every day, more than once per week, once per week, one to three times per month, or never. Each activity category was assigned a score based on frequency, with higher scores indicating greater frequency. These scores were then weighted according to intensity. Frequency of mild activity per day was calculated as: 3 + times/week = 1, 1–2 times/week = 0.43, 1–2 times/month = 0.14, <1/month = 0.07, never = 0. Moderate activities weighted ×2, vigorous activities weighted ×3. The total values of mild, moderate, and vigorous activities were added up to create a scale of physical activities. This measure has been validated in previous research (26).

Smoking status was categorised as non-Smoker included never smoked and former smoker (having consumed at least 100 cigarettes in one’s lifetime), and current smoker. Body mass index (BMI) was self-reported and analysed as a continuous variable.

Healthy ageing, the outcome variable in this study, was operationalised as an aggregate measure encompassing freedom from cognitive impairment, freedom from disability, and high physical functioning. Freedom from cognitive impairment score ≥ 12 (out of 27) on a measure involving immediate and delayed word recall, serial subtraction, and backwards counting.

Freedom from disability is the ability to perform six activities of daily living ADL (e.g., dressing) and five instrumental activities of daily living IADL (e.g., managing money) without difficulty. ADL included Walking, dressing, bathing or showering, eating, getting in/out of bed, using the toilet. IADL included: using the telephone, managing money, taking medication, shopping for grocery, preparing a hot meal.

High physical functioning ≤ 1 difficulty performing 11 tasks (e.g., climbing stairs). Tasks included walking one block, walking several blocks, sitting for about 2 h, getting up from a chair after sitting for long periods, climbing one flight of stairs without resting, climbing several flights of stairs without resting, stooping, kneeling, or crouching, reaching or extending arms above shoulder level, pulling or pushing large objects like a living room chair, lifting or carrying weights over 10 pounds, like a heavy bag of groceries, picking up a dime from a table. For each of these variables’ individuals were categorised as healthy or not healthy.

Finally, participants were classified as “healthy” if they met all three criteria; otherwise, they were classified as “not healthy” (27). This dichotomous specification reflects an established operationalisation of HA in longitudinal studies using the Health and Retirement Study (HRS), facilitating comparability with previous research (27, 28).

Although HA is a multidimensional concept that may include social and environmental domains, the present study focuses on cognitive, physical, and functional aspects of healthy ageing. Social participation was therefore conceptualised separately as an upstream social determinant that may influence HA trajectories rather than as a component of the outcome measure. This approach aligns with life course and social determinants of health perspectives, which recognise the role of social environments and engagement in shaping functional ability and wellbeing in later life. While the WHO framework emphasises the importance of maintaining functional ability through interactions between individuals and their environments, the current operationalisation captures key functional domains of HA while examining social participation as a potential pathway influencing these outcomes.

### Statistical analysis

Descriptive analyses were first conducted to examine the distribution of all study variables across each wave of data collection. Structural Equation Modeling (SEM) was employed to explicitly test longitudinal direct and indirect pathways linking socioeconomic factors, social participation, health related behaviours, and HA. The analysis was carried out using Mplus Version 8.11 (49), with maximum likelihood estimation used for model parameter estimation. Model fit was evaluated using the chi-square statistic (χ^2^), Comparative Fit Index (CFI), Tucker–Lewis Index (TLI), and Root Mean Square Error of Approximation (RMSEA).

The model specified a latent variable representing baseline socioeconomic status (SES), indicated by income and wealth measured in 2006. Direct effects of SES and social participation at baseline (2006) on HA at follow-up (2016) were estimated, alongside indirect effects operating through health-related behaviours (smoking and physical activity) and BMI assessed in 2010. The final model estimated HA at the 2016 follow-up while adjusting for baseline demographic covariates, including age, gender, race, marital status, and educational attainment. To account for prior health status and longitudinal changes in HA, HA measured in both 2006 and 2010 was included as an additional covariate, independent of demographic adjustment. Standardised estimates, standard errors, and confidence intervals were reported to facilitate interpretation of the magnitude and precision of the estimated associations.

## Results

The analysis included 9,748 participants with complete data in all included waves. Participants with missing data were excluded to allow for complete case analysis. A depiction of participants included and the causes of attrition throughout the study can be found in Figure 1. The average age of participants in 2006 was 68.66 years (SD = 10.52 years). The percentage of individuals over 50 who achieved HA in 2016 was 37.08%, a decrease from 37.14% in 2010 and 38.09% in 2006. In 2006, 87.57% of individuals reported engaging in social participation. Wealth distribution in 2006 showed 12.43% in the lowest bracket, and 35.21% in the highest bracket. Income showed 9.99% in the lowest, and 47.73% in the highest income brackets. Description of variables can be found in Table 1.

**Figure 1:**
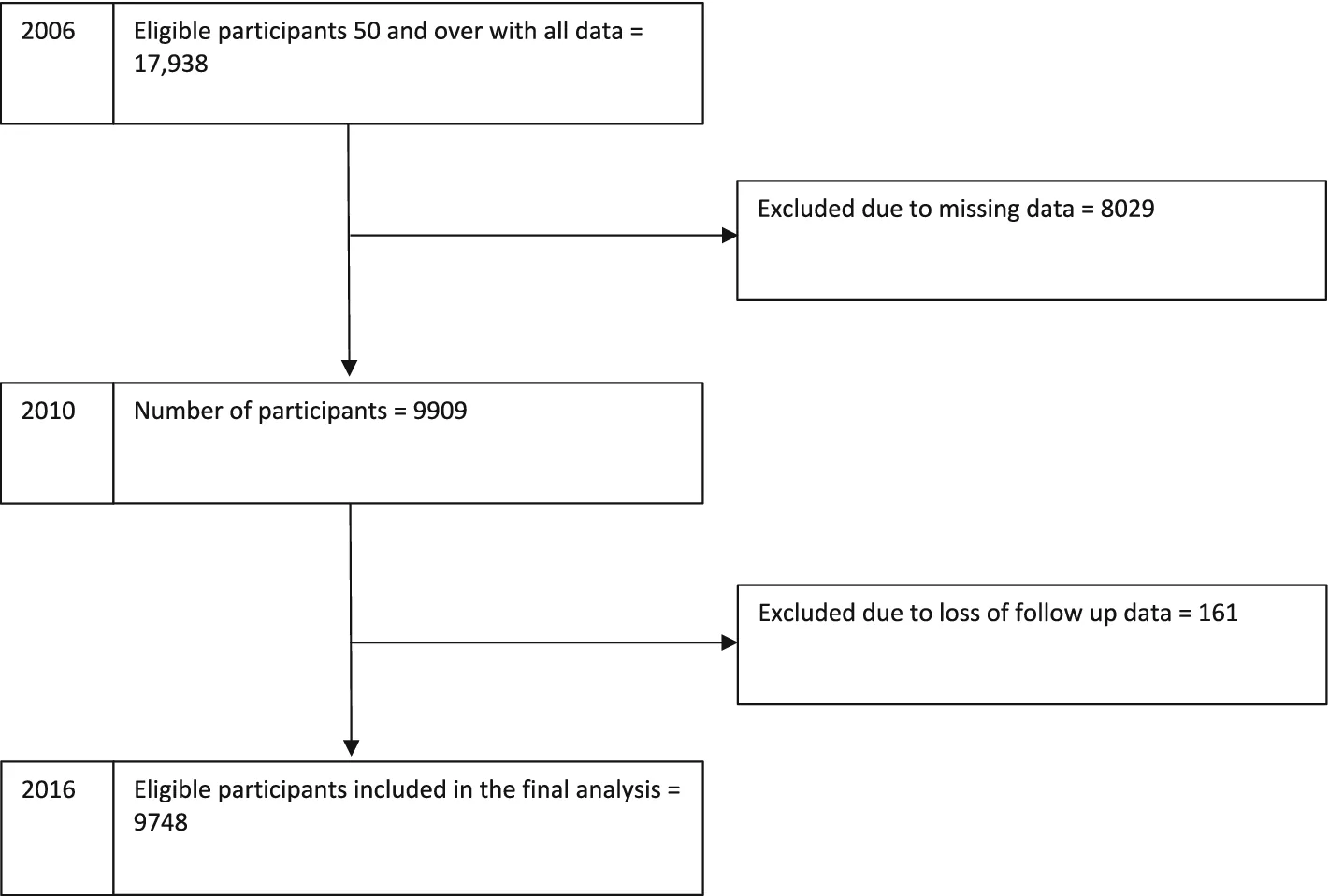
Flowchart including the number of participants in the analysis (HRS-USA 2006–2010-2016).

**Table 1:** Characteristics of participants included in the analysis in each wave (HRS-USA 2006–2010-2016) (*n* = 9,748).

Year	Variables		Mean/Percentage/SD
	Gender	Male	41.66%
		Female	58.34%
	Race	Black	13.89%
		Non-Black	86.11%
	Education	<Highschool	22.67%
		Highschool/Equivalent	52.45%
		Some college	4.31%
		College and above	20.57%
2006			
	Age (mean)		68.66 ± 10.93
	HA		38.09%
	Social participation		87.57%
	Marital status	Married	76.00%
		Unmarried/Divorced	15.92%
		Widowed	8.08%
	Wealth	Lowest	12.43%
		Second lowest	21.13%
		Second highest	31.23%
		Highest	35.21%
	Income	Lowest	9.99%
		Second lowest	15.87%
		Second highest	26.41%
		Highest	47.73%
2010			
	Physical activity (mean)		11.40 ± 6.21
	Body mass index (BMI) (mean)		30.11 ± 6.01
	Healthy ageing		37.14%
	Smoking	Nonsmoker	90.43%
		Smoker	9.57%
2016			
	HA (outcome)		37.08%

SEM was applied to assess the relationships among SES, social participation, health behaviours, and their association with HA (Table 2). The analysis examined both direct and indirect associations between socioeconomic factors, social participation, and HA. The direct effects assessed the immediate relationships between variables, such as how socioeconomic position and social participation directly affected HA outcomes. Social participation in 2006 showed a direct positive, significant association with HA in 2016 (estimate = 0.09, 95% CI: 0.08, 0.11), indicating that greater social engagement in mid-life was associated with better ageing outcomes later.

**Table 2:** Standardised path coefficients from structural equation modelling of socioeconomic factors, social participation, health behaviours, and healthy ageing in older Americans (HRS 2006–2010–2016, *n* = 9,748).

Variables		Estimate	95% CI	*p*-value
Socioeconomic factors 2006 (Latent Variable)				
Poverty income ratio		0.99	(0.99,0.99)	<0.001
Wealth		0.98	(0.98,0.98)	<0.001
Socioeconomic factors 2006				
Age 2006		0.98	(0.97,0.98)	<0.001
Smoking 2010				
Socioeconomic factors 2006		0.97	(0.97,0.97)	<0.001
Social participation 2006		0.01	(0.01,0.02)	<0.001
Physical activity 2010				
Socioeconomic factors 2006		0.02	(0.02,0.03)	<0.001
Social participation 2006		0.84	(0.83,0.84)	<0.001
BMI 2010				
Socioeconomic factors 2006		0.98	(0.98,0.99)	<0.001
Social participation 2006		−0.00	(−0.01,0.02)	NS
HA 2016				
2006	Socioeconomic factors	0.03	(−0.07, 0.14)	NS
	Age	0.02	(−0.03,0.06)	NS
	Marital status	−0.01	(−0.02,0.00)	NS
	Social participation	0.09	(0.08,0.11)	<0.001
	Gender	0.00	(−0.01,0.01)	NS
	Education	0.01	(0.00,0.02)	NS
	Race	0.12	(0.11,0.14)	<0.001
	HA	−0.15	(−0.15,0.02)	NS
2010	Smoking	0.00	(−0.04,0.04)	NS
	BMI	−0.03	(−0.09,0.02)	NS
	Physical activity	0.31	(0.29,0.33)	<0.001
	HA	0.46	(0.45, 0.47)	<0.001
Model fit				
RMSEA		0.08		<0.001
CFI		0.92		
TLI		0.90		

Table presents the standardized estimates, 95% confidence intervals (CI), and associated *p*-values from the Structural Equation Model (SEM) analysing the relationships between baseline socioeconomic and social factors (2006), intermediate health behaviours (2010), and the outcome HA (2016). RMSEA, Root Mean Square Error of Approximation; CFI, Comparative Fit Index; TLI, Tucker–Lewis Index. Continuous variables (age, physical activity, BMI) were entered as continuous measures; therefore, no reference categories apply. Categorical variables were coded as follows: smoking status (current smoker vs non-smoker [reference]), Social participation was coded as yes versus no (reference category) gender (female vs male [reference]), race (non-Black vs Black [reference]), and marital status (not married vs married/partnered [reference]). Estimates for categorical variables represent the difference between the specified category and the reference category.

SES was not directly associated with HA but showed associations with health behaviours that were subsequently associated with HA. Indirect effects were evaluated by considering how SES and social participation impacted behaviours such as smoking, physical activity, and BMI, which in turn affected HA. Higher socioeconomic status was linked to greater physical activity (estimate = 0.02, 95% CI: 0.02, 0.03), which showed better ageing outcomes. However, an unexpected positive association was observed between higher SES and smoking, with individuals of higher socioeconomic status showing a greater likelihood of smoking. This finding contrasts with the commonly reported inverse socioeconomic gradient in smoking behaviour and is explored further in the Discussion. Social participation was primarily associated with increased physical activity (estimate = 0.84, 95% CI: 0.83, 0.84), which played a key role in enhancing HA. Thus, the analysis showed that the pathways to HA were not only shaped by direct effects but also indirectly through health behaviour pathways.

The commonly accepted criteria indicate acceptable model fit when RMSEA values are ≤0.08, and CFI and TLI values are ≥0.90, with values ≥0.95 indicating good fit (29). In the present study, the model demonstrated acceptable fit (RMSEA = 0.08, CFI = 0.92, TLI = 0.90), meeting the recommended thresholds for SEM.

Figure 2 illustrates the longitudinal associations between SES, social participation, health behaviours, and HA in 2016, arrows highlight both the direct and indirect pathways testing associations with HA outcomes.

**Figure 2:**
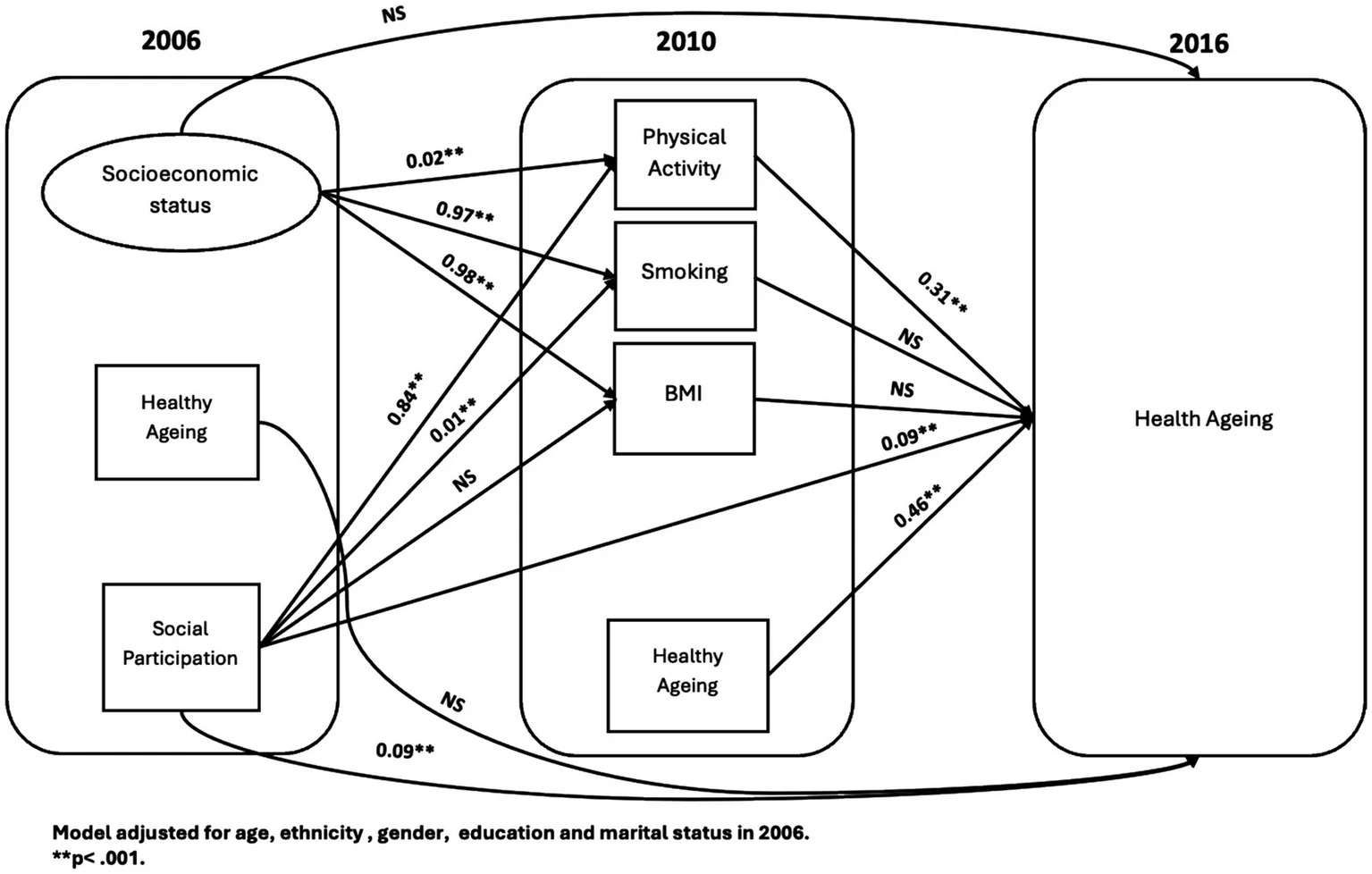
Path diagram illustrating the direct and indirect pathways influencing healthy ageing (HRS-USA 2006–2010-2016) (*n* = 9,748).

To further validate the findings, two sensitivity analyses were conducted. The first involved excluding the social participation variable. When this was done, education, a socioeconomic indicator, emerged as a significant exposure, with individuals possessing higher educational attainment demonstrating better HA outcomes. The second sensitivity analysis included only participants who had achieved HA at baseline (2006), excluding any individuals who were unhealthy at that time. The findings from the sensitivity analysis aligned with the original findings (Supplementary Table S1).

## Discussion

This study examined the longitudinal associations between social participation, socioeconomic factors, health behaviours, and HA among older Americans. Findings presented that greater social participation was positively associated with better HA outcome. Additionally, higher socioeconomic status was associated with increased physical activity which in turn showed positive HA outcomes. Social participation also had an indirect effect on HA, primarily through its effect on physical activity, which was significantly linked to better ageing outcomes. The hypothesis that suggested that social participation and socioeconomic factors are associated with HA was partially confirmed. Increased social participation and higher SES were associated with HA both directly and indirectly by shaping health behaviours.

The analysis found that greater SES was associated with a higher possibility of engaging in greater levels of physical activity. This result aligns with previous research that shows higher socioeconomic conditions are associated with greater participation in sports and physical activities (30). Socioeconomic disadvantage presents multiple barriers such as limited access to resources and facilities, safety concerns, and a lack of social support, all of which reduce opportunities and motivation for physical activity. These challenges often result in lower leisure-time physical activity and persist into older age, contributing to health inequalities. Therefore, understanding how SES influences physical activity participation is critical for developing accessible, socially supportive, and culturally appropriate interventions for older adults (31–34).

Conversely, higher SES was associated with a higher probability of smoking, which contrasts with the typical trend where individuals with higher SES tend to smoke less. This unexpected pattern may be explained by several factors. The life-course and social mobility framework, as demonstrated by Avila et al. (35), highlights that older adults who experienced either upward or downward SES mobility, or who remained consistently disadvantaged were significantly less likely to quit smoking at 65 years compared to those with persistently high SES. This indicates that early life socioeconomic disadvantage may continue to influence smoking cessation behaviour in later life, even among individuals who attain higher SES later in life. This influence may help explain the unexpected positive association between adult SES and smoking prevalence found in our study.

As previous studies concluded Low SES contributes to heavier disease burden (13) therefore, they are more likely to go through Teachable moments which are defined as naturally occurring life or health events that enhance an individual’s motivation to adopt healthier behaviours or decrease engagement in risky activities (13). Concluding that lower SES individuals may quit smoking earlier due to the health complications associated with smoking, as they have a higher likelihood of experiencing chronic illnesses (36, 37).

The relationship among socioeconomic status and BMI has also been well-established in recent studies, which found that individuals with greater socioeconomic status tend to have significantly higher BMI levels compared to those from lower socioeconomic backgrounds (38, 39).

Older adults reporting social participation were more likely to be smokers and to participate in physical activity. These results agree with a study suggesting that enhancing social support networks, especially from family, could play a critical role in promoting physical activity among older adults (40).

Health behaviours, such as physical activity was a key factor influencing HA outcomes in this study. Physical activity was found to have a positive association with HA at final follow up. The results align with well-established research showing the long-term health benefits of physical activity. Regular participation in physical activity was associated with higher HA Index scores, indicating that older adults who stay active tend to experience better health outcomes (41). These outcomes are supported by a systematic review and meta-analysis by Daskalopoulou et al. (42). Their analysis revealed a strong positive association between physical activity and HA, showing that individuals who were physically active had significantly higher odds of leading a healthier life in older age compared to their inactive counterparts.

The positive associations between social participation and HA in final follow up underscore the importance of early social engagement in promoting better health outcomes as individuals age. This finding supports previous studies suggesting that early social engagement is vital for maintaining physical and mental health in later life (43). The results also align with existing literature that highlights the role of social participation in enhancing wellbeing and reducing the risk of physical and mental health decline in older adults (44).

Demographic factors, including age race and marital status were tested in the model. All were nonsignificant except for race that played a role in predicting HA. Inequalities in health outcomes across ethnic groups are well documented. Consistent with the current findings that Black individuals in the US experience accelerated or poorer ageing compared to their Non-Black counterparts, Macias-Konstantopoulos et al. (45) concluded that Black populations endure disproportionate burdens of chronic diseases, comorbidities, and premature mortality. The Northern Manhattan Study (NOMAS) also reported that Hispanic and Black participants had a higher prevalence of mild cognitive impairment (MCI) and dementia compared to White individuals (46). The current results therefore align with a rising amount of evidence advocating for structural changes and health equity initiatives as essential components for optimising ageing trajectories across racial groups.

### Strengths

Building on these findings, a key strength of this study is in its longitudinal design, which enables the examination of temporal relationships and the tracking of changes over time. This approach is particularly valuable for understanding how socioeconomic conditions and social participation influence health behaviours and HA. The sample size further strengthens the study, providing a more generalisable representation of the population. Additionally, the study adds to the current literature by testing associations that have not been sufficiently explored before, particularly the role of social participation in shaping health behaviours and ultimately HA. By demonstrating the significant associations of social participation on behaviour, this study offers valuable insights that could inform interventions aimed at promoting healthier ageing across diverse populations.

This study’s findings align with the Rowe and Kahn model of successful ageing (25) the WHO framework for HA (50, 51) and the biopsychosocial model (47), which underscore that HA is a multidimensional process shaped by biological, psychological, social, and environmental influences (48). Physical activity and social participation, central to Rowe and Kahn’s model, were linked to healthier ageing outcomes. However, the study reveals limitations in Rowe and Kahn model of successful ageing and the emphasis on ideal physical health, showing that individuals with higher BMI or a history of smoking can still age healthily through social and behavioural engagement. The WHO model is more comprehensively supported, as the results underscore the role of socioeconomic status, social participation, and health behaviours in influencing functional ability and well-being. The longitudinal design of the study further supports the WHO’s life course approach, highlighting the importance of early and sustained interventions for HA.

### Limitations

The study is limited by its reliance on self-reported measures, which may be subject to recall bias and social desirability bias, whereby participants provide responses they perceive to be socially acceptable rather than entirely accurate. This may have particularly affected self-reported measures such as physical activity and body mass index (BMI).

This is an inherent issue in large national surveys, where participants may not always accurately remember or report their behaviours. This study experienced participant attrition across the waves, which is a common challenge in longitudinal research. Also, excluding missing data ensured complete case analysis, it may reduce the sample size and could potentially introduce bias if the missing data are not completely at random.

## Conclusion

The findings from this study highlight the multifaceted determinants of HA, including socioeconomic factors, social participation, and health behaviours. A particularly important insight from this study is the positive effect of social participation on HA, suggesting that social participation plays a crucial role in shaping ageing trajectories. These findings add to the expanding evidence on determinants of HA and highlight the importance of considering social participation in assessments of ageing outcomes. Policies and interventions aimed at improving socioeconomic conditions, promoting physical activity, and fostering social engagement could play an important role in improving the quality of life for older adults.

Approaches such as supporting volunteering and social activity initiatives, developing age friendly neighbourhoods, and improving local environments to enhance safety and accessibility for older adults may help reduce social isolation and create opportunities for sustained engagement across the life course. Future research should evaluate the effectiveness of social engagement interventions.

## Data Availability

Publicly available datasets were analyzed in this study. This data can be found at: https://hrs.isr.umich.edu/.
