# Supplementary material for: The relationship between socioeconomic factors, social participation, behaviours, and healthy ageing: an analysis of longitudinal data
Source: Front Public Health. 2026 Sep 30;14:1932979. doi: 10.3389/fpubh.2026.1932979 (PMC13661375; doi:10.3389/fpubh.2026.1932979)
Supplement: Supplementary file 1 [file Supplementary_file_1.DOCX]

## **Sensitivity analysis (Excluding Social Participation)**

To test the robustness of the findings, a sensitivity analysis was conducted using an alternative model specification, in which social participation was excluded (table 1). The results indicated that the latent socioeconomic variable remained well defined, with both wealth (estimate = 0.98, 95% CI: 0.98–0.98) and poverty income ratio (estimate = 0.99, 95% CI: 0.99–0.99) continuing to load strongly and significantly. Socioeconomic position at baseline significantly predicted health behaviours at Wave 10, including smoking (estimate = 0.97, 95% CI: 0.97–0.97, p < 0.001), physical activity (estimate = 0.13, 95% CI: 0.12–0.15, p < 0.001), and BMI (estimate = 0.98, 95% CI: 0.98–0.99, p < 0.001).

Importantly, once social participation was removed from the model, education emerged as a significant predictor of Healthy Ageing in 2016 (estimate = 0.02, 95% CI: 0.01–0.03, p < 0.001). Individuals with higher levels of educational attainment demonstrated more favourable healthy ageing outcomes, underscoring the independent role of education as a socioeconomic determinant of later life health. Ethnicity was also positively associated with healthy ageing (estimate = 0.20, 95% CI: 0.19–0.22, p < 0.001), while age, sex, marital status, and baseline healthy ageing were not statistically significant.

At Wave 10, physical activity continued to exert a strong direct effect on healthy ageing (estimate = 0.40, 95% CI: 0.38–0.42, p < 0.001), and healthy ageing measured at Wave 10 strongly predicted healthy ageing outcomes at Wave 13 (estimate = 0.57, 95% CI: 0.55–0.58, p < 0.001). Smoking and BMI at Wave 10 were not significantly associated with healthy ageing.

The results reinforce the importance of socioeconomic indicators, particularly education, and highlight that the exclusion of social participation shifts the explanatory power towards other socioeconomic determinants.

Table 1. Path coefficient estimates using SEM (HRS-USA 2006–2010-2016) (n =9748).

| **Variables** | | **Estimate** | **95% CI** | ***p*-Value** |
| --- | --- | --- | --- | --- |
| Socioeconomic factors **2006** (Latent Variable) | | | | |
|  | Poverty income ratio | 0.99 | (0.99,0.99) | <0.001 |
|  | Wealth | 0.98 | (0.98,0.98) | <0.001 |
| Socioeconomic factors **2006** | | | | |
|  | Age 2006 | 0.98 | (0.97,0.98) | <0.001 |
| Smoking **2010** | | | | |
|  | Socioeconomic factors **2006** | 0.97 | (0.97,0.97) | <0.001 |
| Physical Activity **2010** | | | | |
|  | Socioeconomic factors **2006** | 0.13 | (0.12,0.15) | <0.001 |
| BMI **2010** | | | | |
|  | Socioeconomic factors **2006** | 0.98 | (0.98,0.99) | <0.001 |
| Healthy ageing **2016** | | | | |
| **2006** | Socioeconomic factors | 0.04 | (-0.09, 0.17) | NS |
|  | Age | 0.02 | (-0.03,0.07) | NS |
|  | Marital status | -0.01 | (-0.02,0.00) | NS |
|  | Sex | -0.00 | (-0.01,0.01) | NS |
|  | Education | 0.02 | (0.01,0.03) | <0.001 |
|  | Ethnicity | 0.20 | (0.19,0.22) | <0.001 |
|  | Healthy ageing | -0.02 | (-0.06,0.02) | NS |
|  | | | | |
| **2010** | Smoking | 0.00 | (-0.05,0.05) | NS |
|  | BMI | -0.04 | (-0.11,0.03) | NS |
|  | Physical activity | 0.40 | (0.38,0.42) | <0.001 |
|  | Healthy ageing | 0.57 | (0.55,0.58) | <0.001 |
|  | | | | |

## **Sensitivity Analysis (Baseline Healthy Sample)**

A second sensitivity analysis was conducted to further validate the main findings by restricting the sample to participants who were classified as healthy at baseline (2006), thereby excluding individuals who did not meet the healthy ageing criteria at that time (Table 2). The analytic sample for this model included 3,874 participants. The latent variable representing baseline socioeconomic status remained well defined, with strong and statistically significant loadings for poverty income ratio (0.99, 95% CI: 0.99–0.99, p < 0.001) and wealth (0.98, 95% CI: 0.98–0.98, p < 0.001).

Baseline socioeconomic factors predicted subsequent health behaviours at Wave 10, including smoking (0.98, 95% CI: 0.97–0.98, p < 0.001), physical activity (0.07, 95% CI: 0.05–0.09, p < 0.001), and BMI (0.98, 95% CI: 0.98–0.98, p < 0.001). Social participation at baseline exhibited moderate associations with physical activity (0.59, 95% CI: 0.57–0.60) but was not significantly associated with smoking or BMI.

For Healthy Ageing outcomes in 2016, social participation at baseline was a significant positive predictor (0.24, 95% CI: 0.20–0.25, p < 0.001), and ethnicity was also positively associated with healthy ageing (0.24, 95% CI: 0.21–0.27, p < 0.001). Other baseline variables, including socioeconomic factors, age, marital status, Sex, and education, were not significantly associated with healthy ageing. At Wave 10, physical activity continued to have a strong positive effect on healthy ageing (0.32, 95% CI: 0.30–0.35, p < 0.001), whereas smoking and BMI were not significantly associated with the outcome.

Overall, the results closely mirrored those of the original analysis, confirming the robustness of the main model’s findings. This sensitivity analysis demonstrates that among participants already classified as healthy at baseline, social participation and physical activity remained key predictors of long-term healthy ageing, highlighting the importance of maintaining engagement in social and health promoting behaviours for sustained health outcomes.

Table 2. Path coefficient estimates using SEM (HRS-USA 2006–2010-2016) (n =3874).

| **Variables** | | **Estimate** | **95% CI** | ***p*-Value** |
| --- | --- | --- | --- | --- |
| Socioeconomic factors **2006** (Latent Variable) | | | | |
|  | Poverty income ratio | 0.99 | (0.99,0.99) | <0.001 |
|  | Wealth | 0.98 | (0.98,0.98) | <0.001 |
| Socioeconomic factors **2006** | | | | |
|  | Age 2006 | 0.97 | (0.97,0.97) | <0.001 |
| Smoking **2010** | | | | |
|  | Socioeconomic factors **2006** | 0.98 | (0.97,0.98) | <0.001 |
|  | Social participation **2006** | 0.01 | (0.00,0.01) | NS |
| Physical Activity **2010** | | | | |
|  | Socioeconomic factors **2006** | 0.07 | (0.05,0.09) | <0.001 |
|  | Social participation **2006** | 0.59 | (0.57,0.60) | NS |
| BMI **2010** | | | | |
|  | Socioeconomic factors **2006** | 0.98 | (0.98,0.98) | <0.001 |
|  | Social participation **2006** | 0.00 | (-0.01,0.01) | NS |
| Healthy ageing **2016** | | | | |
| **2006** | Socioeconomic factors | -0.19 | (-0.44, 0.06) | NS |
|  | Age | -0.00 | (-0.10,0.10) | NS |
|  | Marital status | 0.03 | (-0.07,0.13) | NS |
|  | Social participation | 0.24 | (0.20,0.25) | <0.001 |
|  | Sex | -0.01 | (-0.03,0.01) | NS |
|  | Education | -0.02 | (-0.04,0.00) | NS |
|  | Ethnicity | 0.24 | (0.21,0.27) | <0.001 |
|  | | | | |
| **2010** | Smoking | 0.13 | (0.01,0.25) | NS |
|  | BMI | 0.10 | (-0.03,0.22) | NS |
|  | Physical activity | 0.32 | (0.30,0.35) | <0.001 |
|  | | | | |
